# INDIVIDUAL AND ORGANIZATIONAL BENEFITS OF CO-LOCATING NURSING HOME AND CHILD CARE SERVICES

**DOI:** 10.1093/geroni/igae098.3168

**Published:** 2024-12-31

**Authors:** Helen Wang, Shannon Jarrott, Mengyao Ma

**Affiliations:** University of Michigan, Ann Arbor, Michigan, United States; The Ohio State University, Columbus, Ohio, United States; Hong Kong University of Science and Technology, Hong Kong, Hong Kong

## Abstract

Co-location nursing homes with childcare services has gained recognition for fostering positive intergenerational relationships. Researchers have identified benefits to individual participants, but little attention has been paid to organizational outcomes. Using econometrics methods, we analyzed historical data of all 19,641 unique US nursing homes from 2015Q1 to 2020Q1 to assess the association between co-location and nursing home quality measures, including deficiency reports and resident performance measures. Leveraging Centers for Medicare & Medicaid Services (CMS) and Data Axle data, our empirical model tested the association between on-site childcare and nursing home quality. We controlled for potential confounds, including nursing home characteristics (e.g., capacity and ownership), regional socioeconomic status, and year and zip code fixed effects. Combining business location data and public information sources, we identified 139 nursing homes with on-site childcare services operating in our sample period. Regression analyses indicated that nursing homes with on-site childcare had 9.94% lower yearly deficiency case reports than homes without childcare (p < 0.01) due to lower health deficiency rates but not fire safety deficiencies. Considering quarterly resident and institution measures, nursing homes with on-site childcare centers demonstrated 1.1% to 5.8% higher resident vaccination rates (p < 0.01) and significantly lower psychotropic medication use (p < 0.001) than those without childcare. Our study highlights that co-locating childcare and nursing homes is associated with better nursing home performance, benefiting residents and organizations. Further research should address mechanisms by which outcomes are achieved. Intergenerational program co-location may support benefits beyond those experienced by younger and older participants.

